# The emergence of socioeconomic inequalities in smoking during adolescence and early adulthood

**DOI:** 10.1186/s12889-023-16182-w

**Published:** 2023-07-18

**Authors:** Joana Alves, Julian Perelman, Elisabete Ramos, Anton E Kunst

**Affiliations:** 1 NOVA National School of Public Health, Public Health Research Centre, Comprehensive Health Research Center, CHRC, NOVA University Lisbon, Avenida Padre Cruz, Lisbon, 1600-560 Portugal; 2 Department of Public Health and Forensic Sciences, and Medical Education, Faculty of Medicine, University of Porto, Alameda Prof. Hernâni Monteiro, Porto, 4200-319 Portugal; 3 EPIUnit - Institute of Public Health, University of Porto, Rua das Taipas 135, Porto, 4050-091 Portugal; 4 Department of Public and Occupational Health, Amsterdam Public Health research institute, Amsterdam UMC, University of Amsterdam, Meibergdreef 9, Amsterdam, 1105 AZ The Netherlands

**Keywords:** Socioeconomic inequalities, Smoking history, Young adults, Adolescents, Longitudinal analysis

## Abstract

**Background:**

While it is known that educational inequalities in smoking start during early and middle adolescence, it is unknown how they further develop until adulthood. The aim of this article is to map, in the Portuguese context, how educational inequalities in smoking emerge from pre-adolescence until young adulthood.

**Methods:**

This study used longitudinal data from the EPITeen Cohort, which recruited adolescents enrolled in schools in Porto, Portugal. We included the 1,038 participants followed at ages 13 (2003/2004), 17, 21, and 24 years. We computed the odds ratio (OR) for the prevalence of smoking states (never smoking, experimenter, less-than-daily, daily and former smoker) and the incidence of transitions between these states, as function of age and education, stratified by sex. We also added interaction terms between age and education.

**Results:**

Educational inequalities in daily smoking prevalence, with higher prevalence among those with lower educational level, emerged at 17 years old and persisted until higher ages. They were formed in a cumulative way by the increased risk of experimenting between 13 and 17 years, and increased risk of becoming daily smoker between 17 and 21 years. The incidence of smoking cessation was higher among the higher educated. Inequalities were formed similarly for women and men, but with lower level and showed no significance among women.

**Conclusions:**

These results highlight that actions to prevent smoking should also take in account the potential impact in smoking inequalities, and should focus not only on middle adolescence but also on late adolescence and early adulthood.

**Supplementary Information:**

The online version contains supplementary material available at 10.1186/s12889-023-16182-w.

## Introduction

Despite the notable progress in tobacco control, smoking is still an important issue worldwide. Although smoking rates fell 15% globally, from 2007 to 2017, currently, 1.1 billion people smoke, and at least 40% have already attempted to quit [1]. However, the use of tobacco is not uniformly distributed across the population strata.

Socioeconomic inequalities in smoking are often described, with most studies reporting a strong association between education and smoking [2]. Less educated men are more likely to smoke in most countries worldwide, while this gradient is less marked among women [3, 4]. The educational differences have generally been widening in recent decades, mainly due to a larger decline in smoking prevalence among the higher educated [5]. Even now, educational inequalities in smoking contribute substantially to inequalities in mortality [6].

Smoking usually starts before the age of 18, with 15% of smokers starting before 15 years old [7]. Educational differences in smoking are already observed among adolescents, although with differences across considered birth cohorts, sexes, and countries [8]. Smoking is more common among early adolescents (ages 10–14) and late adolescents (ages 15–21) whose parents have lower educational levels [9], and especially among adolescents with lower personal educational levels [10, 11]. Gradients have been found to persist until early adulthood (21–24 years old), with higher prevalence of ever-smoking among the lower educated compared to the higher educated [12].

Although educational inequalities in smoking are found to appear during adolescence, there is still uncertainty regarding the specific ages at which most of these educational inequalities emerge. A precise identification of the most important ages and corresponding smoking transitions is important for targeting interventions addressing those inequalities. For example, interventions focused exclusively on secondary schools might miss opportunities if most of inequalities in smoking are formed at about 17 years or older.

Two studies have described the emergence of educational inequalities across adolescence and early adulthood. One of them, a 10-year longitudinal study in the United States, found evidence that inequalities begin in early adolescence, around 12–14 years old, and widen thereafter [13]. A study among adolescents born in 1970 in Britain found relatively stable inequalities between 16 and 26 years old. These inequalities emerged from persistent inequalities in smoking initiation up to 26 years old, while there were no inequalities in smoking cessation until that age [14]. Beyond those two longitudinal studies, other have investigated the emergence of educational inequalities using retrospective questions about smoking initiation and/or cessation. Those studies suggested that educational differences in smoking emerged during adolescence and widened until early adulthood [8, 15]. Recall bias might be a problem in these studies, since the participants may not remember accurately the timing of events. Longitudinal studies limit the risk of recall bias by instead presenting the questions at the time participants join the study.

The aim of this study is to add to the existing body of literature, using a longitudinal study that allows an accurate assessment of how educational inequalities in smoking evolve from early adolescence until young adulthood. This is of crucial importance since they would be a marker of cumulative socioeconomic disadvantage in health later in live. Given the usual differences on smoking patterns between men and women in Portugal, the analysis is stratified by sex.

## Materials and methods

### Study design and population

This study uses a sample comprising adolescents from the Epidemiological Health Investigation of Teenagers in Porto, Portugal (EPITeen). The study’s methods are already described elsewhere [16] and summarized here. The information was obtained through self-administered questionnaires. The participants were followed across four waves: 2003/2004, 2007/2008, 2011/2013, and 2014/2015; being on average 13 years old, 17 years old, 21 years old, and 24 years old, at the respective waves. In the first wave, all adolescents born in 1990 and enrolled in public and private schools in Porto were invited to participate (2,786). Of those, 2,159 agreed to participate (77.5%). In the second wave, 1,716 participants (79.5%) were re-evaluated, and a further 783 adolescents were newly included into the cohort as they moved to the schools of Porto. In the third and fourth study waves, 1,764 and 1,094 participants were re-evaluated, respectively. We considered only those who had participated in at least three of the four waves, including the last wave. The final sample was composed of 1,038 individuals. The attrition rate in this study was 39%.

The EPITeen Cohort was approved by the Portuguese Commission for Data Protection, and the Ethics Committees of Hospital S. João and of Instituto de Saúde Pública da Universidade do Porto (ISPUP). Written informed consent was obtained from parents and adolescents in the first and second waves, and from participants in the remaining waves. The study met the guidelines for protection of human subjects concerning their safety and privacy.

### Measures

For each wave, we computed smoking prevalence rates. Respondents were classified as never smokers (never experienced cigarette smoking until that wave), ever experimenters (have experimented with smoking at some point, but have not smoked regularly until that wave), less-than-daily smokers (smoked cigarettes at the time of that wave, but less-than-daily), daily smokers (smoked cigarettes daily at the time of that wave), and former smokers (reported smoking daily or less-than-daily in the past wave, but no longer smoked at the time of that wave).

Incidence rates were calculated based on the transition in smoking states between the waves. For each wave we used the data to measure the incidence rates of experimenting (number of new experimenters in that wave as a proportion of the non-smokers in the previous wave), less-than-daily smoking (number of new less-than-daily smokers in the wave as proportion of those who did not smoke, experimented smoking, smoked daily, or had quit smoking in the previous wave), daily smoking (number of new daily smokers in the wave among those who do not smoke, experimented smoke, smoked less-than-daily, or had quit smoking in the previous wave), and former smoking (number of new former smokers in the wave amongst those who smoked daily or less-than-daily in the previous wave). As a result, events between the waves were not considered.

For the participants with no information about smoking status in one of the three first waves, we re-constructed the tobacco history using the age of smoking initiation as reported in the next wave, by taking in consideration both the age of smoking initiation and the smoking status reported in the following waves (more detail in appendix 2). We performed a sensitivity analysis in which we repeated the estimations for original variables without the information given by the age of smoking initiation. There were no noteworthy changes in the results, except that 95% confidence intervals were wider (appendix 3).

We measured acquired education in young adulthood based on the completed level of education of the participant in the last wave. We distinguished between high education (university degree or tertiary education) and low education (otherwise). We considered the highest degree completed for those participants who were still enrolled in school at the time of the last wave(34.8%).

### Statistical analysis

We modelled whether the smoking behaviour was associated with educational attainment, in two steps.

We computed the prevalence and incidence of smoking, using generalized estimating equation (GEE) models with a binomial distribution and an identity link, with fixed effects for survey year, and interaction terms for age with education. We stratified the analysis by sex. This method was chosen since we were modelling longitudinal binary outcome variables for never smoker, experimenter, smoke less-than-daily, smoke daily, and former smoker (yes = 1 and no = 1). GEE is a method designed to model longitudinal data across time within the same individual, and it is usually used with non-normal data, such as binary data. It allowed us to make inferences about the population, accounting for the within-subject correlation. To fit a model, we must specify both the family and the link function [17]. We used this method to graph the evolution of the marginal effects for prevalence and incidence rates.

We also computed the prevalence and incidence of smoking, as function of education and age, stratified by sex, using GEE assuming a binomial distribution and a logit link. This method allowed us to compute the odds ratio for longitudinal binary response data [17]. We repeated this procedure, adding interactions for age with education.

All analyses were conducted in Stata version 13.0, using xtgee command.

## Results

The number of participants was 1,038, 49.3% of which were men. Most respondents were never smokers at 13 years old: 81.3% among boys and 75.1% among girls (Table 1). This prevalence fell to 19.5% and 29.7% among 24-year-old men and women, respectively. From the 13 years old to the 24 years old, the percentage of daily smokers increased from 0.2 to 32.6% among men, and from 1.0 to 20.5% amongst women. The incidence of experimentation increased between 13 and 21 years old, from 17.2 to 35.5% among men, and from 23.0 to 28.8% among women. The incidence of daily smoking increased greatly between 17 and 21 years old, reaching 22.5% amongst men and 17.3% among women.

**Table 1:** Prevalence and incidence of smoking per 100 persons, per age (EPITeen Cohort, 2003, 2007, 2011, and 2014)

Prevalence	13 years old	17 years old	21 years old	24 years old
Men				
Never smoker	81.3	53.9	23.4	19.5
Experimenter	17.2	33.4	36.9	33.8
Smoke less-than-daily	1.4	6.1	11.3	7.0
Smoke daily	0.2	6.3	27.2	32.6
Former smoker	0.0	0.4	1.2	7.0
**Women**				
Never smoker	75.1	54.8	32.5	29.7
Experimenter	23.0	32.9	36.1	36.3
Smoke less-than-daily	1.0	4.4	7.6	5.3
Smoke daily	1.0	7.8	22.4	20.5
Former smoker	0.0	0.2	1.3	8.2
**Incidence**	**< 13 years old**	**13–17 years old**	**17–21 years old**	**21–24 years old**
**Men**				
Experimenter ^(1)^	17.2	26.2	35.5	13.3
Less-than-daily smoker ^(2)^	1.4	5.9	10.2	4.4
Daily smoker ^(3)^	0.2	6.1	22.5	11.0
Former smoker ^(4)^	0.0	25.0	7.9	15.7
**Women**				
Experimenter ^(1)^	23.0	22.3	28.8	7.6
Less-than-daily smoker ^(2)^	1.0	3.8	6.6	3.3
Daily smoker ^(3)^	1.0	7.1	17.3	4.2
Former smoker ^(4)^	0.0	10.0	10.9	22.8

*Legend:* (1) Number of experimenters among the never smokers in the previous wave. (2) Number of less-than-daily smokers among the non-less-than-daily smokers in the previous wave. (3) Number of daily smokers among the non-daily smokers in the previous wave. (4) Number of former smokers among those who smoke in the previous wave

**Fig. 1:**
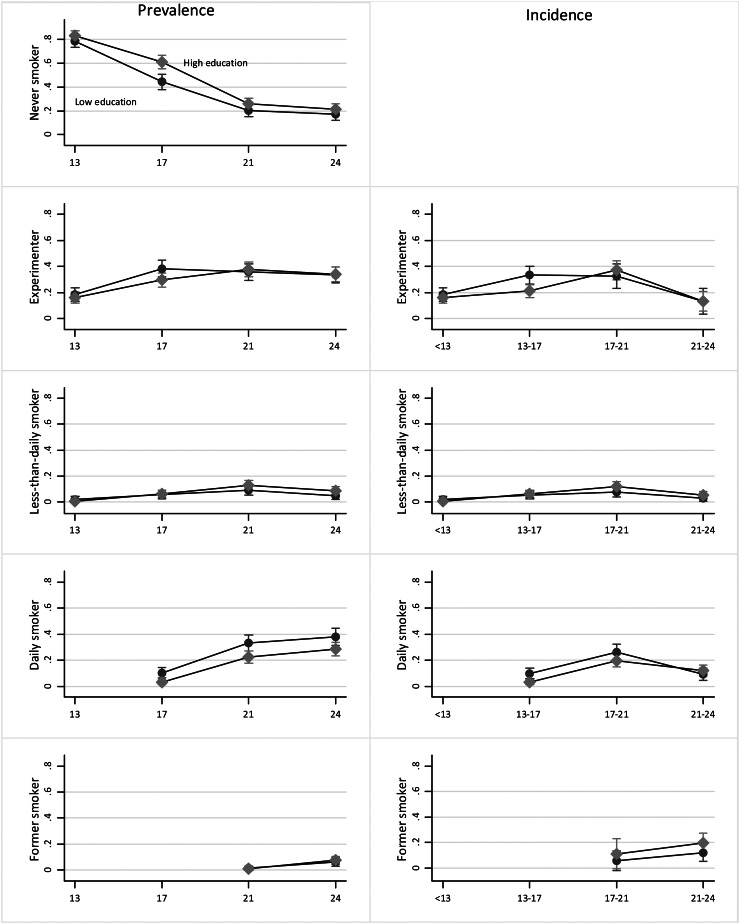
**Trends in the prevalence and incidence of smoking among men by education level (EPITeen cohort 2003, 2007, 2011, and 2014)** Legend: Marginal effects for each age given the education category, from the generalized estimation equation

Figure 1 presents the prevalence and incidence of smoking among men. Inequalities in the prevalence of never smoking were present at all ages. There was an increased risk of experimenting between 13 and 17 years old among the lower educated. Regarding daily smoking, inequalities emerged at 17 years old and persisted.

**Fig. 2:**
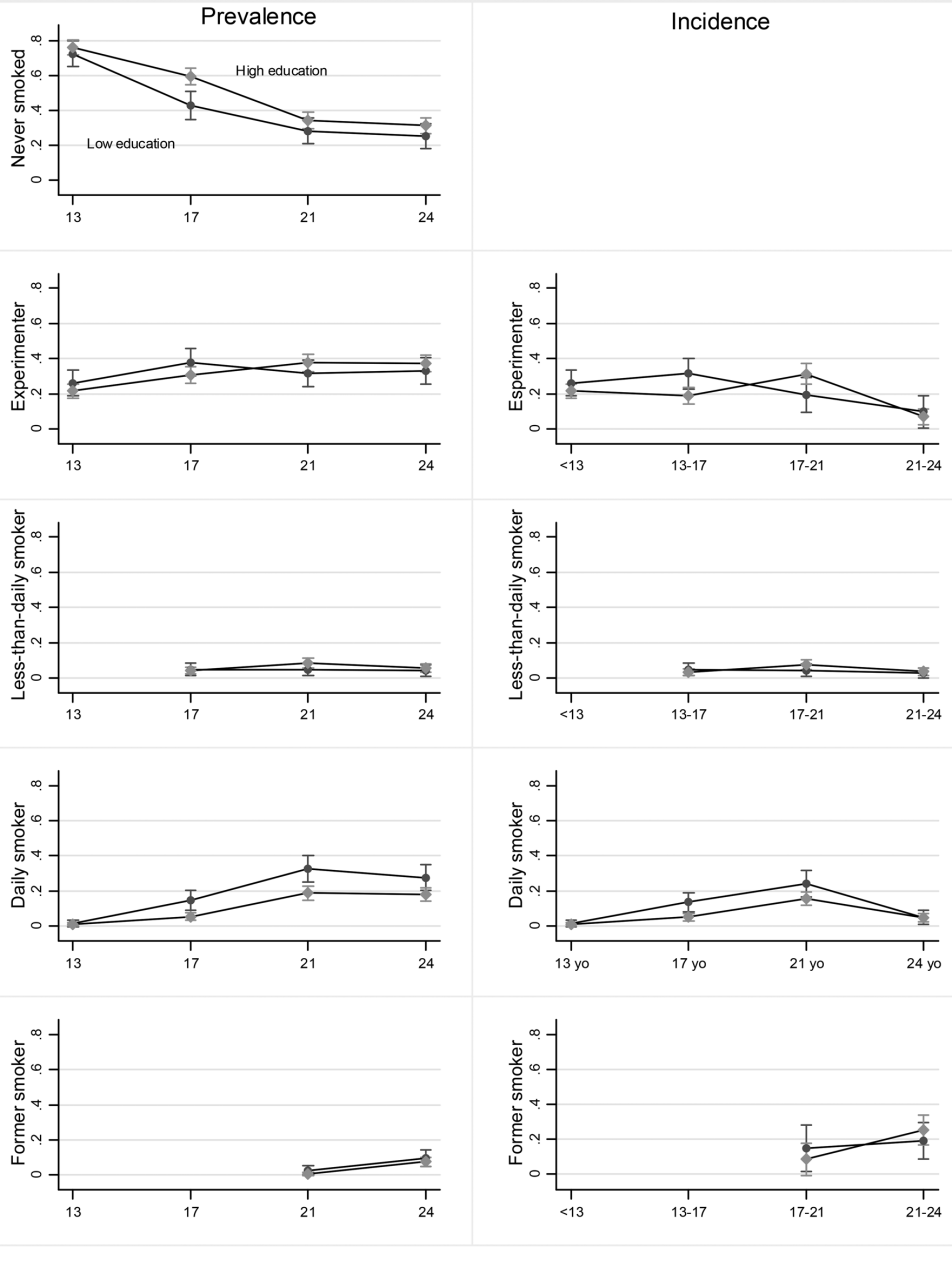
**Trends in the prevalence and incidence of smoking among women by education level (EPITeen cohort 2003, 2007, 2011, and 2014)** Legend: Marginal effects for each age given the education category, from the generalized estimation equation

Among women (Fig. 2), inequalities in the prevalence of never smoking were present at all ages. Inequalities in daily smoking prevalence emerged at 17 years old and persisted. The lower educated were more likely to experiment until 17 years old and to be daily smokers at all ages after 17 years old.

Estimates based on regression analyses are presented in Table 2 (prevalence) and Table 3 (incidence). Regarding prevalence, higher educated men were more likely to be never smokers at all ages (Odds Ratio (OR) was 1.63 [1.19; 2.24]); the differences were greatest at 17 years old (OR for the interaction age/education was 1.52 [1.00; 2.30]) (Table 2). The inequalities in daily smoking prevalence existed at all ages among men (OR = 0.62 [0.46; 0.82], being more noticeable at 17 years old (OR for the interaction with 17 years and education was 0.42 [0.21; 0.85]). Among women, the higher educated were more likely to be never smokers at all ages (OR = 1.63 [1.19; 2.24]), and the differences were greatest at 17 years old (OR for the interaction was 1.48 [1.02; 2.14]). The higher educated women were less likely to experiment at 17 years old (OR for the interaction was 0.60 [0.38; 0.95]. Higher educated women were less likely to be daily smokers at all ages (OR = 0.49 [0.35; 0.69]).

**Table 2:** Odds ratio for the smoking prevalence from the GEE, with family binomial and logit link, and stratified by sex (EPITeen cohort, 2003, 2007, 2011, 2014)

	Model 1		Model 2							
	Higher education^1^		Higher educ. x 13 years old ^(2)^		Higher educ. x 17 years old ^(2)^		Higher educ. x 21 years old ^(2)^		Higher educ. x 24 years old ^(2)^	Test for interaction
Men										
Never smoker	1.63	[1.19;2.24]	1.02	[0.65;1.61]	1.52	[1.00;2.30]	1.04	[0.67;1.62]	1.00	0.12
Experimenter	0.90	[0.70;1.16]	0.83	[0.49;1.38]	0.66	[0.42;1.03]	1.07	[0.68;1.67]	1.00	0.15
Less-than-daily	1.37	[0.91;2.05]	0.17	[0.03;0.97]	0.59	[0.22;1.59]	0.84	[0.34;2.06]	1.00	0.20
Daily smoker	0.62	[0.46;0.82]	NE		0.42	[0.21;0.85]	0.88	[0.59;1.30]	1.00	0.06
Former smoker	1.19	[0.59;2.36]	NE		NE		0.63	[0.14;2.90]	1.00	0.55
Women										
Never smoker	1.54	[1.09;2.18]	0.92	[0.62;1.36]	1.48	[1.02;2.14]	0.99	[0.68;1.45]	1.00	0.05
Experimenter	1.00	[0.74;1.34]	0.64	[0.40;1.04]	0.60	[0.38;0.95]	1.08	[0.68;1.71]	1.00	0.03
Less-than-daily	1.42	[0.79;2.57]	NE		0.61	[0.19;1.94]	1.32	[0.43;4.02]	1.00	0.39
Daily smoker	0.49	[0.35;0.69]	0.99	[0.18;5.45]	0.57	[0.30;1.10]	0.84	[0.50;1.39]	1.00	0.40
Former smoker	0.75	[0.39;1.47]	NE		NE		0.36	[0.09;1.50]	1.00	0.16

*Legend:* Model 1 = Odds ratio for GEE for smoking variables adjusting for ages and education level. Model 2 = Model 1 adding the interactions for age with education. NE = Could not be estimated due to small number of population at risk. ^(1)^ the reference category is lower education. ^(2)^ Reference category. 95% confidence intervals in square brackets

**Table 3:** Odds ratio for the smoking incidence from the GEE, with family binomial and logit link (EPITeen cohort, 2003, 2007, 2011, 2014)

	Model 1		Model 2							
	Higher education^1^		Higher educ. x 13 years old ^(2)^		Higher educ. x 17 years old ^(2)^		Higher educ. x 21 years old ^(2)^		Higher educ. x 24 years old ^(2)^	Test for interaction
Men										
Experimenter	0.80	[0.61;1.04]	0.85	[0.26;2.75]	0.53	[0.17;1.73]	1.22	[0.36;4.06]	1.00	0.12
Less-than-daily	1.34	[0.89;2.01]	0.16	[0.02;1.07]	0.59	[0.17;2.06]	0.87	[0.27;2.79]	1.00	0.23
Daily smoker	0.68	[0.49;0.93]	NE		0.22	[0.08;0.64]	0.54	[0.24;1.21]	1.00	0.02
Former smoker	1.86	[0.89;3.87]	NE		NE		1.17	[0.16;8.72]	1.00	0.88
Women										
Experimenter	0.81	[0.61;1.08]	1.13	[0.30;4.18]	0.74	[0.20;2.82]	2.77	[0.67;11.40]	1.00	0.03
Less-than-daily	1.31	[0.75;2.27]	NE		0.59	[0.14;2.54]	1.45	[0.34;6.17]	1.00	0.39
Daily smoker	0.36	[0.76;1.18]	0.58	[0.07;4.57]	0.33	[0.10;1.16]	0.59	[0.19;1.90]	1.00	0.33
Former smoker	1.18	[0.58;2.42]	NE		NE		0.37	[0.06;2.10]	1.00	0.26

*Legend:* Model 1 = Odds ratio for GEE for smoking variables adjusting for ages and education level. Model 2 = Model 1 adding the interactions for age with education. NE = Could not be estimated due to small number of population at risk. ^(1)^ the reference category is lower education. ^(2)^ Reference category. 95% confidence intervals in square brackets

The incidence of daily smoking (Table 3) was higher among low educated men at all ages (OR = 0.68 [0.49; 0.93]); and the differences were higher for 13–17 years old (OR for the interaction was 0.22 [0.08; 0.64]). Among women, the differences in smoking incidence according to education were not significant.

## Discussion

### Key results

This article sought to map how educational inequalities in smoking evolve from pre-adolescence until young adulthood, using a Portuguese longitudinal study. We found that inequalities in daily smoking prevalence emerged at 17 years old and persisted until higher ages. They were formed in a cumulative way by the increased risk of experimenting between 13 and 17 years, and increased risk of becoming daily smoker between 17 and 21 years. Inequalities were formed along similar pathways for women and men, but with lower level and showed no significance among women.

### Potential limitations

The use of a longitudinal study design allows us to observe the trends of inequalities from adolescence into early adulthood. However, some potential problems should be considered. First, this design is subject to attrition over time (with a 39% attrition rate), which might result in a selective study population.

Second, the percentage of persons that had at least one missing answer about smoking in at least one survey was 38% (either because they refused to answer or because they were not included in that specific wave). We aimed to overcome this problem by using the age of smoking initiation and the age of trying the first cigarette indicated in the next wave, in order to create a smoking history for participants lacking one response in the first three surveys. The sensitivity analyses (not shown for the sake of brevity) allow us to conclude that the strategy did not significantly affect the results.

Third, around 35% of the participants were still enrolled in education at the time of the last questionnaire (24 years old), and were classified according to the highest completed education instead of the currently attended education. This potential misclassification might result in an underestimation of the differences in smoking between the participants with high versus low education.

### Interpretation of results

We may question how smoking in adolescence is linked to education achieved in young adulthood. A possible explanation is unobserved heterogeneity, which means that there are some underlying causes affecting simultaneously the early smoking behaviour and the pathways to educational attainment. For example, people with greater future orientation may be more willing to invest in education and might be more inclined to protect themselves from the health hazards of smoking [18]. Another example is educational and societal aspirations, since they will influence future academic achievement and have been previously associated with smoking in adolescence and adulthood [19]. Also, cognitive functioning influences how information is perceived, while potentially interfering with the perception of smoking hazards [18].

We found a higher incidence of experimentation between 13 and 17 years old by those with lower education. This association might have different explanations. For example, adolescents with negative school experiences might be more prone to relate themselves with deviant groups that are more likely to smoke [20] and they may initiate smoking in order to cope with higher school pressure and demands [21]. In addition, restrictions on sales to minors might be more weakly enforced in the poorest communities. Weak enforcement of sales-related policies in Portugal facilitate adolescents’ access to cigarettes and increased visibility of smoking [22].

The results also showed that young adults with lower academic achievement were more likely to become daily smokers, compared to the ones with higher academic achievement. This might be explained by the fact that the young adults starting a job have their own money, also they are more exposed to smoking co-workers, or their peers, which make them more likely to reinforce their smoking habits [23]. Another explanation is that those attaining highest education levels are more likely tend to quit after experimenting, and avoid becoming addicted [15].

The inequalities in smoking incidence emerged before the age of 18 among men, while among women, the inequalities in smoking emerged and widened across all age ranges, although not significantly. Our results suggest that relevant transitions to adulthood may differ across sex, for example due to cultural norms regarding smoking. In addition, this difference could also reflect that Portuguese women are at an earlier stage of the smoking epidemic, with more marked initiation among low educated women across all ages [24]. It is important to reinforce that the lack of statistical significance in women is not due to a lack of power, since the prevalence of tobacco use is similar in men and women over the waves.

Previous studies have showed that socioeconomic inequalities in adolescent smoking are associated to family background and indicators of their perceived family socioeconomic status [25]. Additionally, evidence suggests that the transmission of smoking behaviour between parents and their children is likely to be consistent across social strata, but parental smoking habits are usually socioeconomically patterned [26]. Therefore, parental smoking influence could potentially contribute to the perpetuation of socioeconomic disparities in smoking. While our objective was not to explore the role of parental smoking and socioeconomic status in their children’s smoking, we acknowledge that these factors may be significant contributors to smoking inequalities among offspring, and future studies could investigate their role.

## Conclusions

Inequalities in smoking were formed in a cumulative way, from adolescence to early adulthood, by the increased risk of experimenting between 13 and 17 years, and increased risk of becoming daily smoker between 17 and 21 years, among those with lower educational levels. These results highlight that the initiatives to prevent smoking should also take in account the potential impact in smoking inequalities, and should focus not only on middle adolescence but also on late adolescence and early adulthood. Also, given that most research to date has been focussed on early and middle adolescence, our results stress the need for additional research on inequalities in smoking initiation in late adolescence and early adulthood. Declarations.

## Electronic supplementary material

Below is the link to the electronic supplementary material.

Supplementary Material 1

Supplementary Material 2

Supplementary Material 3

## Data Availability

The datasets analysed during the current study are not publicly available, since it could compromise research participant privacy, but are available from the corresponding author on reasonable request, and with permission of EPIUnit - Institute of Public Health, University of Porto.

## List of Abbreviations

ISPUP: Instituto de Saúde Pública da Universidade do Porto
GEE: Generalized Estimating Equation Models
OR: Odds Ratio
95%CI: 95% Confidence Intervals
NE: Could not be estimated due to small number of participants at risk
