# Supplementary material for: The emergence of socioeconomic inequalities in smoking during adolescence and early adulthood
Source: BMC Public Health. 2023 Jul 18;23:1382. doi: 10.1186/s12889-023-16182-w (PMC10354878; doi:10.1186/s12889-023-16182-w)
Supplement: Supplementary file 1 — Supplementary Material 1 [file 12889_2023_16182_MOESM1_ESM.docx]

Appendix 1: Descriptive statistics, per age (EPITeen Cohort, 2003, 2007, 2011, and 2014).

|  | **Women n (%)** | | | | **Men n (%)** | | | |
| --- | --- | --- | --- | --- | --- | --- | --- | --- |
|  | **Low education** | | **High education** | | **Low education** | | **High education** | |
| **Total sample** | **145** | **(27.6)** | **380** | **(72.4)** | **221** | **(43.2)** | **291** | **(64.7)** |
| **Smoking Prevalence** |  |  |  |  |  |  |  |  |
| **Age 13** |  |  |  |  |  |  |  |  |
| Never smoker | 105 | (72.4) | 290 | (76.3) | 174 | (78.7) | 242 | (83.2) |
| Experimenter | 38 | (26.2) | 82 | (21.6) | 41 | (18.6) | 47 | (16.2) |
| Smoke less-than-daily | 0 | (0.0) | 5 | (1.3) | 5 | (2.3) | 2 | (0.7) |
| Smoke daily | 2 | (1.4) | 3 | (0.8) | 1 | (0.5) | 0 | (0.0) |
| Former smoker | 0 | (0.0) | 0 | (0.0) | 0 | (0.0) | 0 | (0.0) |
| **Age 17** |  |  |  |  |  |  |  |  |
| Never smoker | 62 | (42.8) | 226 | (59.5) | 98 | (44.3) | 178 | (61.2) |
| Experimenter | 55 | (37.9) | 117 | (30.8) | 85 | (38.5) | 86 | (29.6) |
| Smoke less-than-daily | 7 | (4.8) | 16 | (4.2) | 13 | (5.9) | 18 | (6.2) |
| Smoke daily | 21 | (14.5) | 20 | (5.3) | 23 | (10.4) | 9 | (3.1) |
| Former smoker | 0 | (0.0) | 1 | (0.3) | 2 | (0.9) | 0 | (0.0) |
| **Age 21** |  |  |  |  |  |  |  |  |
| Never smoker | 41 | (28.3) | 130 | (34.2) | 45 | (20.4) | 75 | (25.8) |
| Experimenter | 46 | (31.7) | 143 | (37.6) | 79 | (35.8) | 110 | (37.8) |
| Smoke less-than-daily | 7 | (4.8) | 33 | (8.7) | 20 | (9.1) | 38 | (13.1) |
| Smoke daily | 47 | (32.4) | 71 | (18.7) | 74 | (33.5) | 65 | (22.3) |
| Former smoker | 4 | (2.8) | 3 | (0.8) | 3 | (1.4) | 3 | (1.0) |
| **Age 24** |  |  |  |  |  |  |  |  |
| Never smoker | 37 | (25.3) | 119 | (31.3) | 38 | (17.2) | 62 | (21.3) |
| Experimenter | 48 | (33.1) | 142 | (37.4) | 74 | (33.5) | 99 | (34.0) |
| Smoke less-than-daily | 6 | (4.1) | 22 | (5.8) | 11 | (5.0) | 25 | (8.6) |
| Smoke daily | 40 | (27.6) | 68 | (17.9) | 84 | (38.0) | 83 | (28.5) |
| Former smoker | 14 | (9.7) | 29 | (7.6) | 14 | (6.3) | 22 | (7.6) |
| **Smoking Incidence** |  |  |  |  |  |  |  |  |
| **<13 years old** |  |  |  |  |  |  |  |  |
| Experimenter | 38 | (26.2) | 82 | (21.6) | 41 | (18.6) | 47 | (16.2) |
| Less-than-daily smoker | 0 | (0.0) | 5 | (1.3) | 5 | (2.3) | 2 | (0.7) |
| Daily smoker | 2 | (1.4) | 3 | (0.8) | 1 | (0.5) | 0 | (0.0) |
| Former smoker | 0 | (0.0) | 0 | (0.0) | 0 | (0.0) | 0 | (0.0) |
| **13 -17 years old** |  |  |  |  |  |  |  |  |
| Experimenter | 33 | (31.4) | 55 | (19.0) | 58 | (33.3) | 51 | (21.1) |
| Less-than-daily smoker | 7 | (4.8) | 13 | (3.5) | 12 | (5.6) | 18 | (6.2) |
| Daily smoker | 19 | (13.3) | 18 | (4.8) | 22 | (10.0) | 9 | (3.1) |
| Former smoker | 0 | (0.0) | 1 | (12.5) | 2 | (33.3) | 0 | (0.0) |
| **17-21 years old** |  |  |  |  |  |  |  |  |
| Experimenter | 12 | (19.4) | 71 | (31.4) | 32 | (32.7) | 66 | (37.1) |
| Less-than-daily smoker | 6 | (4.4) | 27 | (7.4) | 16 | (7.7) | 33 | (12.1) |
| Daily smoker | 29 | (23.4) | 55 | (15.3) | 52 | (26.3) | 56 | (19.9) |
| Former smoker | 4 | (14.3) | 3 | (8.3) | 2 | (5.6) | 3 | (11.1) |
| **21-24 years old** |  |  |  |  |  |  |  |  |
| Experimenter | 4 | (9.8) | 9 | (6.9) | 6 | (13.3) | 10 | (13.3) |
| Less-than-daily smoker | 4 | (2.9) | 12 | (3.5) | 6 | (3.0) | 14 | (5.5) |
| Daily smoker | 4 | (4.1) | 13 | (4.2) | 14 | (9.5) | 27 | (12.0) |
| Former smoker | 10 | (18.5) | 26 | (25.0) | 11 | (11.7) | 20 | (19.4) |
