# Supplementary material for: The emergence of socioeconomic inequalities in smoking during adolescence and early adulthood
Source: BMC Public Health. 2023 Jul 18;23:1382. doi: 10.1186/s12889-023-16182-w (PMC10354878; doi:10.1186/s12889-023-16182-w)
Supplement: Supplementary file 2 — Supplementary Material 2 [file 12889_2023_16182_MOESM2_ESM.docx]

**Appendix 2. Missing data information**

|  | **1st wave** | **2nd wave** | **3rd wave** | **4th wave** | **Number of  missing data** | **Number of respondents** | **%** |
| --- | --- | --- | --- | --- | --- | --- | --- |
| Excluded | 🗶 | 🗶 | 🗶 | 🗶 | 4 | 60 | 2% |
|  | 🗶 | 🗶 | 🗶 | ✓ | 3 | 4 | 0% |
|  | 🗶 | 🗶 | ✓ | 🗶 | 3 | 13 | 0% |
|  | 🗶 | 🗶 | ✓ | ✓ | 2 | 5 | 0% |
|  | 🗶 | ✓ | 🗶 | 🗶 | 3 | 345 | 12% |
|  | 🗶 | ✓ | 🗶 | ✓ | 2 | 26 | 1% |
|  | 🗶 | ✓ | ✓ | 🗶 | 2 | 228 | 8% |
|  | ✓ | 🗶 | 🗶 | 🗶 | 3 | 290 | 10% |
|  | ✓ | 🗶 | 🗶 | ✓ | 2 | 10 | 0% |
|  | ✓ | 🗶 | ✓ | 🗶 | 2 | 67 | 2% |
|  | ✓ | ✓ | 🗶 | 🗶 | 2 | 425 | 14% |
|  | ✓ | ✓ | ✓ | 🗶 | 1 | 431 | 15% |
| **Sum of excluded** |  |  |  |  |  | **1904** | **65%** |
| Considered | 🗶 | ✓ | ✓ | ✓ | 1 | 281* | 10% |
|  | ✓ | 🗶 | ✓ | ✓ | 1 | 52* | 2% |
|  | ✓ | ✓ | 🗶 | ✓ | 1 | 37* | 1% |
|  | ✓ | ✓ | ✓ | ✓ | 0 | 668 | 23% |
| **Sum of considered** |  |  |  |  |  | **1038** | **35%** |

**Legend**: * completed using information about age of smoking initiation.
