# Supplementary material for: The emergence of socioeconomic inequalities in smoking during adolescence and early adulthood
Source: BMC Public Health. 2023 Jul 18;23:1382. doi: 10.1186/s12889-023-16182-w (PMC10354878; doi:10.1186/s12889-023-16182-w)
Supplement: Supplementary file 3 — Supplementary Material 3 [file 12889_2023_16182_MOESM3_ESM.docx]

**Appendix 3. Sensitivity analysis**

Odds ratio for the smoking prevalence from the GEE, with family binomial and logit link, and stratified by sex (EPITeen cohort, 2003, 2007, 2011, 2014).

|  | **Model 1** | **Model 2** | | | | |
| --- | --- | --- | --- | --- | --- | --- |
|  | **Higher education^1^** | **Higher educ. x 13 years old ^(2)^** | **Higher educ. x 17 years old ^(2)^** | **Higher educ. x 21 years old ^(2)^** | **Higher educ. x 24 years old ^(2)^** | **Test for  interaction** |
| Men |  |  |  |  |  |  |
| Never smoker | 1.24 [0.91; 1.70] | 0.56 [0.35; 0.89] | 0.87 [0.55; 1.39] | 0.91 [0.55; 1.52] | 1.00 | 0.02 |
| Experimenter | 0.97 [0.76; 1.25] | 0.55 [0.29; 1.04] | 0.66 [0.40; 1.08] | 0.93 [0.57; 1.51] | 1.00 | 0.14 |
| Less-than-daily | 1.40 [0.93; 2.10] | 0.17 [0.03; 0.97] | 0.60 [0.22; 1.67] | 0.89 [0.36; 2.20] | 1.00 | 0.20 |
| Daily smoker | 0.63 [0.47; 0.85] | NE | 0.49 [0.21; 1.10] | 0.90 [0.59; 1.35] | 1.00 | 0.23 |
| Former smoker | 1.16 [0.56; 2.41] | NE | NE | 2.74 [0.34; 22.46] | 1.00 | 0.35 |
| Women |  |  |  |  |  |  |
| Never smoker | 1.29 [0.90; 1.84] | 0.84 [0.59; 1.21] | 1.38 [0.95; 1.99] | 1.03 [0.70; 1.51] | 1.00 | 0.03 |
| Experimenter | 0.99 [0.74; 1.33] | 0.77 [0.45; 1.33] | 0.62 [0.39; 1.01] | 1.27 [0.78; 2.06] | 1.00 | 0.03 |
| Less-than-daily | 1.42 [0.79; 2.57] | NE | 0.61 [0.19; 1.94] | 1.32 [0.43; 4.02] | 1.00 | 0.39 |
| Daily smoker | 0.51 [0.36; 0.72] | 0.99 [0.18; 5.49] | 0.54 [0.27; 1.08] | 0.89 [0.53; 1.49] | 1.00 | 0.36 |
| Former smoker | 0.71 [0.36; 1.39] | NE | NE | 0.63 [0.17; 2.35] | 1.00 | 0.49 |

**Legend**: Model 1 = Odds ratio for GEE for smoking variables adjusting for ages and education level. Model 2 = Model 1 adding the interactions for age with education. NE = Could not be estimated due to small number of population at risk. ^(1)^ the reference category is lower education. ^(2)^ Reference category. 95% confidence intervals in square brackets.

Odds ratio for the smoking incidence from the GEE, with family binomial and logit link (EPITeen cohort, 2003, 2007, 2011, 2014).

|  | **Model 1** | **Model 2** | | | | |
| --- | --- | --- | --- | --- | --- | --- |
|  | **Higher education^1^** | **Higher educ. x 13 years old ^(2)^** | **Higher educ. x 17 years old ^(2)^** | **Higher educ. x 21 years old ^(2)^** | **Higher educ. x 24 years old ^(2)^** | **Test for  interaction** |
| Men |  |  |  |  |  |  |
| Experimenter | 0.77 [0.57; 1.05] | 0.83 [0.30; 2.32] | 0.93 [0.36; 2.43] | 1.30 [0.48; 3.49] | 1.00 | 0.67 |
| Less-than-daily | 1.35 [0.89; 2.04] | 0.19 [0.03; 1.26] | 0.78 [0.23; 2.65] | 1.08 [0.35; 3.33] | 1.00 | 0.28 |
| Daily smoker | 0.66 [0.47; 0.92] | NE | 0.37 [0.12; 1.11] | 0.63 [0.29; 1.35] | 1.00 | 0.19 |
| Former smoker | 1.51 [0.71; 3.19] | NE | NE | 3.96 [0.36; 43.69] | 1.00 | 0.26 |
| Women |  |  |  |  |  |  |
| Experimenter | 0.80 [0.57; 1.13] | 1.09 [0.44; 2.68] | 0.69 [0.29; 1.68] | 2.03 [0.78; 5.25] | 1.00 | 0.04 |
| Less-than-daily | 1.35 [0.77; 2.36] | NE | 0.61 [0.14; 2.64] | 1.58 [0.37; 6.66] | 1.00 | 0.36 |
| Daily smoker | 0.57 [0.39; 0.83] | 0.76 [0.10; 5.58] | 0.43 [0.14; 1.37] | 0.92 [0.32; 2.60] | 1.00 | 0.34 |
| Former smoker | 1.15 [0.56; 2.37] | NE | NE | 0.85 [0.17; 4.30] | 1.00 | 0.84 |

**Legend**: Model 1 = Odds ratio for GEE for smoking variables adjusting for ages and education level. Model 2 = Model 1 adding the interactions for age with education. NE = Could not be estimated due to small number of population at risk. ^(1)^ the reference category is lower education. ^(2)^ Reference category. 95% confidence intervals in square brackets.
